# Extract of a Letter from the Rev. Dr. Booker, of Dudley, to a Friend in Gloucestershire, on the Subject of the Cow-Pock

**Published:** 1804-05-01

**Authors:** 


*Extract of a Letter from the* Rev. Dr. Booker, *of Dudley, to a Friend in Gloucestershire, on the Subject of the* Cow-Pock.

EVERY instance of scepticism that I hear of, respecting the efficacy of Vaccine Inoculation, astonishes me nearly as much as if I heard a man deny that the sun is the source of light and heat in the visible creation. Thanks to a beneficent Providence and to Dr. Jenner, that most valuable discovery has triumphed over every species of incredulity throughout the very populous neighbourhood in which I live; where it was at first cautiously received, but is now universally adopted. In my own parish (containing about 14000 inhabitants) so salutary a blessing has it proved, that only ONE VICTIM, *within the last two years*, has been seized by the small-pox; a baneful pest, which, for ages prior to vaccine inoculation, glutted the grave in every region of the earth; but which now only rages and destroys where prejudice or infatuation patronizes and befriends it. Soon would it be no more if these did not now and then furnish it with prey. Were *they*, however, who are actuated by these principles, restricted to select victims from their own children, parental feeling, methinks, agonized by the sufferings and death of those who are *bone of their own bone and flesh of their own flesh*, would speedily cure them of prejudice, or correct their infatuation.

Previous to the introduction of Dr. Jenner's great discovery among my parishioners, I have frequently read the funeral service over seven or eight victims of the small-pox in a day. The single instance of mortality, by the same disorder above mentioned, happened fortunately, I think, for the public. It was deemed an anomaly, an occurrence strange and unnatural by the neighbourhood; and by the parents as an event which *their* prudence might have prevented. Their feelings were, therefore, very acutely affected; fected; no doubt severely charging themselves with being accessary to the death of the child, in not having shielded it from danger by vaccine inoculation.

May no other parent experience such painful reflections! And long may He live to enjoy his feelings, who has been the means, in the hands of Providence, of saving, and whose discovery, under the same Divine Power, will *continue* to save, thousands and tens of thousands of the human race!

*March* 14, 1804.

